# Changes in Body Composition, Basal Metabolic Rate, and Blood Albumin during the First Year following Laparoscopic Mini-Gastric Bypass

**DOI:** 10.1155/2022/7485736

**Published:** 2022-06-28

**Authors:** Adnan Tizmaghz, Mansour Bahardoust, Mostafa Hosseini, Abdulreza Pazouki, Hamidreza Alizadeh Otaghvar, Ghazaal Shabestanipour

**Affiliations:** ^1^General Surgery, Iran University of Medical Sciences, Tehran, Iran; ^2^Department of Epidemiology, School of Public Health, Shahid Beheshti University of Medical Sciences, Tehran, Iran; ^3^Minimally Invasive Surgery Research Center, Rasool -e Akram Hospital, Iran University of Medical Sciences, Tehran, Iran; ^4^General Surgery and Minimally Invasive Surgery, Iran University of Medical Science, Minimally Invasive Research Center, Center of Excellence of European Branch of International Federation for Surgery of Obesity, Tehran, Iran; ^5^General Surgery and Plastic Surgery, Iran University of Medical Science, Minimally Invasive Research Center, Center of Excellence of European Branch of International Federation for Surgery of Obesity, Tehran, Iran; ^6^Private Office, Tehran, Iran

## Abstract

Bariatric surgery is currently the only method that can significantly and continuously reduce weight and improve obesity-related comorbidities in morbidly obese patients. Significant weight loss through bariatric surgery can lead to changes in body composition. This study shows the changes in body composition, basal metabolic rate (BMR), and serum albumin in obese people following bariatric surgery. The study included 880 patients who underwent laparoscopic mini-gastric bypass surgery (LMGBP) between 2016 and 2020. The body mass index (BMI), bioelectrical impedance analysis (BIA), age, gender, blood albumin, WC (waist circumference), HC (hip circumference), BMR, and blood albumin were recorded at 0, 3, 6, and 12 months, postoperatively. The reduction in serum albumin concentration was not consistent with weight loss. Bariatric surgery promotes the breakdown of both fat and lean mass on the arms, torso, and thighs. This size reduction usually aggravates the concomitant skin redundancy in these areas which is a challenge for the plastic surgery team. Interestingly, the rate of lean mass reduction of the arms is faster than that of the torso and thighs. Excessive loss of lean body mass will also lower BMR and lead to subsequent weight gain. Despite the faster loss of proteins and lean mass in somatic areas, internal organs and viscera lose fats faster than proteins. According to this study, visceral proteins are the latest proteins to be affected by weight loss. This finding shows a different metabolic response of viscera comparing to somatic areas.

## 1. Introduction

Overweight or obesity can be defined as excessive accumulation of fat. Treatment options for obesity include nonsurgical (behavioral modification, reducing calorie intake and increasing physical activity, and pharmacological treatment) and surgical (bariatric) treatment. Bariatric surgery can reduce more weight than nonsurgical treatment [1, 2]. In many cases, bariatric surgery can cause nutritional deficiencies. These deficiencies should be detected and corrected as soon as possible to avoid postoperative adverse effects and excessive loss of lean body mass. Besides, the anthropometric values of body weight, skin fold thickness, and average upper arm circumference and biochemical tests can be used to determine the patient's nutritional status. The most commonly used method to measure nutritional status is the serum albumin test, which mainly shows the status of visceral protein synthesis [3–5]. This is very important because excessive protein loss (greater than 25–30% of lean body mass) is totally incompatible with life [6–8]. Skeletal muscle proteins are preferentially depleted acutely after rapid weight loss, whereas visceral proteins remain relatively preserved [6]. Thus, it seems that serum albumin is not a good indicator of lean body mass depletion during rapid weight loss which especially caused by skeletal muscle depletion [6].

The exact effect of weight loss caused by bariatric surgery on energy expenditure and basal metabolic rate (BMR) has not been well quantified especially in patients who underwent laparoscopic mini-gastric bypass (LMGB). The BMR is defined as the minimum amount of energy expenditure compatible with life [9]. Approximately 70% of total energy expenditure per day can be traced back to basic life processes, such as breathing, heart pump, body temperature, and transmission of hormones in the body. Approximately 20% of energy expenditure comes from activity-induced energy expenditure, and another 10% comes from diet-related energy expenditure and food digestion (postprandial heat production) [9].

Malabsorptive procedures such as Roux-en-Y gastric bypass causes more lean mass loss than restrictive procedures (e.g., gastric sleeve resection) [10]. Some possible measures to prevent excessive breakdown of lean muscles are adequate protein intake (at least 1.05 g/kg) [11] and regular physical exercise as early as possible after surgery. Nowadays, bioelectrical impedance analysis (BIA) is a recognized method for assessing body composition, which measures the relationship between body fat and lean body mass after weight loss [12]. Tissues with high water content (muscles, blood vessels, and bones) tend to conduct electricity, while adipose tissue with insufficient water conducts electricity poorly. Body fat percentage is calculated based on a formula that uses measured body water and five factors: electrical resistance, height, weight, age, and gender. There is a linear relationship between bioelectrical impedance index (height2/resistance) and lean mass; since the relative contribution of each segment increases with the body size [13]. Although the effects of LMGB on total body weight have been documented, less is known about changes in bioelectrical impedance indices following LMGB. This study shows the changes in body composition and related events of obese Iranian populations carefully selected in our high-volume bariatric surgery ward.

## 2. Materials and Methods

In this retrospective study, we conducted an analysis of a database maintained by the Obesity Center of Rasool-e-Akram Hospital (a Center of Excellence for Metabolic and Bariatric Surgery, approved by the International Federation for Surgery of Obesity and Metabolic Disorders (IFSO), 2014), Tehran, Iran. The study was approved by the ethical committee of Iran University of Medical Sciences (code: IR.IUMS.rec.1396.16285) and is in accordance with guidelines laid down by the latest version of the Helsinki Declaration.

### 2.1. Study Population

The inclusion criteria were obesity history of more than 5 years; BMI > 40 kg/m^2^ solely or BMI > 35 kg/m^2^ with related comorbidities (such as type 2 diabetes (T2D), hypertension, sleep apnea and other respiratory disorders, nonalcoholic fatty liver diseases, osteoarthritis, lipid abnormalities, gastrointestinal disorders, or heart diseases), history of unsuccessful weight loss attempts, and good motivation for surgery. The patient had to also agree to postoperative diet and exercise. The exclusion criteria were previous history of bariatric surgery or stomach surgery, history of drug abuse, large abdominal hernia, pregnancy or lactation, history of severe mental illness, or BMI > 60 kg/m^2^. Besides, patients who had any of kidney, liver, or gastrointestinal disorders were also excluded. The data of the study participants were retrieved from a database which was filled by trained employees. Also, the patients' records were reassessed by the authors and any irregularities were resolved by direct contact of the patients. Between January 2016 and December 2020, we performed 702 LMGB operations in our department. Of these patients, 419 completed the 12-month follow-up.

### 2.2. Anthropometric Measurements

In this study, we considered demographics and clinical features including age, sex, lifestyle characteristics, laboratory findings, comorbidities, medications, and past medical histories. Weight was measured using a seca scale (seca 700, Hamburg, Germany). The patients were asked to wear thin clothes and no shoes while measuring weight. A Seca stadiometer (Seca 700, Hamburg, Germany) was used to check the participants' height while wearing no shoes. Body mass index (BMI) was calculated as body weight in kilograms, divided by height in meters squared. Waist and hip circumference were measured with light clothes at the top of the iliac crest and the largest part of the buttocks, respectively, using a non-stretchable tape, to the nearest 0.1 cm. The professional segmental body composition analyzer “Tanita BC418” (Tanita BC-418, Tanita Corp., Tokyo, Japan) is used to perform bioelectrical impedance analysis (BIA) for postoperative assessment of changes in body composition. Fat mass (FM), lean mass (LM), muscle and bone mass in trunk, lower and upper extremities, as well as body fat, body fat mass, total body water (TBW), and basal metabolic rate (BMR) were measured. All patients were asked to rest for 30 minutes and fast for at least 10 hours, and then measure by placing four silver electrodes and two detecting electrodes placed at the ulnar aspect of the right wrist and the right medial malleolus. Data on BMI, BIA, weight, height, blood albumin, WC, and HC were collected at baseline, 3, 6, and 12 months, postoperatively.

### 2.3. Statistical Analysis

Data was entered and analyzed with SPSS-22. Data is expressed as mean and standard deviation (SD). The chi-square and repeated measure analysis of variance (ANOVA) test used in the study analysis. Absolute changes in body mass index (BMI), blood albumin, WC, HC, and BIA variables were calculated as the baseline value subtracted from the follow-up values (3, 6, and 12 months). For assessing the relative changes in blood albumin and body composition in response to weight loss, the mean change in BMI, blood albumin, WC, HC, and BIA data during the time of the weight loss (3, 6, and 12 months) were modeled by using a repeated-measure ANCOVA analysis. In this respect we used sex and age as covariates. Statistical significance for all analyses was assumed as *p* ≤ 0.005.

## 3. Results

The study population included 702 obese patients, 568 (80.9%) females and 134 (19.1%) males, with a mean age of 38.9 ± 10.8 years and a mean baseline BMI of 45.9 ± 6.1 kg/m^2^.

### 3.1. Postoperative Changes

The summary data of anthropometrics, BIA, and serum albumin parameters of obese patients and their changes after LMGB surgery (during rapid weight loss) are shown in Tables 1 and 2. As expected, all covariates were reduced from baseline during the postoperative course of weight loss (BMI, body weight, waist circumference, hip circumference, and BIA parameters; free fat mass and body fat) (Tables 1 and 2).

The body composition parameters and their postoperative reduction rate at 12 months after surgery are presented in Table 3. Over the course of postoperative changes, the waist circumference (WC) reduced progressively by −13.8%, −21.7%, and −26.5% in the follow-up points of 3-, 6-, and 12-month, respectively. The hip circumference (HC) was also progressively reduced by −12.3%, −17.8%, and −22.7% in the similar points. In contrast, although drop in albumin concentration was observed early after surgery, the rate of albumin reduction was not consistent with the course of postoperative weight loss (Table 1) and the drop in plasma albumin concentration even began to rise after 6 months despite ongoing weight loss (*p* > 0.05). After one year, the mean albumin value was 4.4 ± 2.7 g/dl. Table 3 also demonstrates reduction in BIA parameters in the course of postoperative follow-up.

## 4. Discussion

In this study, we demonstrated the changes in the body composition, basal metabolic rate, and albumin of the patients who underwent LMGB over a period of one year. According to our results, we observed that except for the albumin, all the parameters were changed significantly after one year follow-up. Protein is the main structural component of human muscles and other tissues. In general, adequate protein is needed to maintain lean muscle mass [8]. Protein deficiency (serum albumin levels below 3 g/dl) associated with bariatric surgery is an unusual and ominous event. Severe protein deficiency can cause swelling (oncotic pressure changes), fatty liver, skin degeneration and hair loss, increasing the severity of infections, muscle atrophy, and increase the risk of bone fractures. Recent studies recommend careful clinical and nutritional follow-up to prevent this rare but potentially dangerous complication through nutritional therapy (nourishing patients with the use of intravenous nutritional substrates for three weeks) or by timely reoperation in more difficult settings [6]. In this study, there was no statistically significant correlation between the blood albumin concentration and the continued loss of muscle protein mass after bariatric surgery. Despite continuous weight loss and negative calorie balance, the albumin concentration in the blood began to increase after 6 months. In addition, since albumin is a negative acute phase protein, its concentration can easily alter in response to infection or inflammation. As a result, inflammatory response may result in low circulating levels of albumin [14, 15]. Albumin has also been criticized as a marker for nutritional assessment due to its lack of specificity and long half-life (about 20 days) [14]. Therefore, blood albumin cannot be a reliable test for evaluating total protein loss after bariatric surgery. As a result, regular measurement of albumin in the early postoperative period is of no use in diagnosing protein deficiency and postoperative malnutrition, which are visceral proteins.

The study showed that BMR was significantly reduced during the follow-up. Compared with the baseline BMR, the BMR after bariatric surgery decreased by 19.9% after 12 months. There is some evidence that weight loss can induce an abnormal reduction in BMR. This reduction in BMR can be attributed to lean mass loss, as lean mass is greatly responsible for variations in energy expenditure at rest. Subsequently, an abnormal low basal metabolic rate may predispose bariatric surgical patients to weight regain [11]. A study by Faria et al. showed that a lower BMR may contribute to weight regain in patients who undergo bariatric surgery. They suggested that the increase in energy expenditure in these patients by increasing the percentage of lean mass in the body and exercise might prevent weight regain [11]. On the contrary to Faria et al.'s impression, Anthanont et al. [16] pointed out that lower BMRs did not contribute to more weight gain than higher BMRs, indicating that habitual differences in food intake or activity are far more effective in causing individual weight gain than BMR. Schiavo et al. stated that cycles of profound weight loss followed by weight regain may result in a significant loss of lean mass (LM) during weight loss and fat mass to lean mass ratio (FM/LM) increase during weight regain, and this may represent a cause of failure or poorer outcome in further bariatric procedures [17]. There are several ways to get an estimate of body composition and its fat and lean masses. Analysis of the body composition plays an important role in nutritional evaluation. The following calculations had formerly been used in the Ardavani, et al.'s [18] study to assess body composition:

  Lean body mass (kg) = total body potassium (mmol)/68.1
  Body fat (kg) = body weight (kg)—free fat mass (kg)

However, in the current study we used BIA for assessing body composition. Postoperative monitoring of waist and hip circumference also showed that waist circumference decreased faster than hip circumference during rapid weight loss. In general, the most pronounced reduction of BMI, FM, and FFM body mass was achieved during the first 3 months following bariatric surgery. This finding is entirely consistent with the previous studies [13, 19, 20].

Currently, there are some other tools developed for assessing malnutrition; the trend of introducing novel tools is based on the minimal use of visceral proteins, such as albumin [21]. Imaging modalities such as computed tomography, ultrasonography, and bioelectrical impedance have been considered as alternatives to the current use of visceral proteins for malnutrition assessment [21]. These approaches may lack deviations that were observed in visceral proteins level in different physiologic and pathologic states.

The strengths of this study were the long-term follow-up, large sample size, and the sophisticated body-composition techniques used. To the best of our knowledge, this is the first independent study on body composition change in Iran after bariatric surgery. Post-bariatric change pattern of blood albumin level has not been recognized before. It might also be helpful in assessing the effectiveness of postoperative strategies. Nevertheless, a longer prospective follow-up, ideally until weight loss stabilization, is needed to better understand the body composition of different anatomic sites and muscle strength, quality, and functional changes after bariatric surgery.

## 5. Conclusion

In conclusion, our results clearly indicate that bariatric surgery and the related accelerated weight loss induce both FM and FFM reduction. Bariatric surgeries reduce both FM and FFM all over the body, with certain areas reducing faster than others.

## Figures and Tables

**Table 1:** Changes of BMI, blood albumin, waist circumference, hip circumference, and bioelectrical impedance analysis ($\bar{x}\pm s$).

Item	Preoperation	3 months postop	6 months postop	12 months postop	*p* value
BMI (kg/m^2^)	**45.6** **±** **6.1**	**35.9** **±** **5.6**	**32.1** **±** **5.1**	**28.9** **±** **4.5**	**p** **<** **0.001**
Albumin (g/dl)	**4.3** **±** **0.42**	**4.2** **±** **0.38**	**4.2** **±** **0.42**	**4.4** **±** **2.7**	*p* **>** 0.05
Waist circumference (cm)	**122.0** **±** **16.9**	**105.6** **±** **11.6**	**95.8** **±** **11.0**	**90.3** **±** **10.8**	**p** **<** **0.001**
Hip circumference (cm)	**137.6** **±** **16.8**	**123.0** **±** **11.9**	**113.9** **±** **10.2**	**107.4** **±** **10.5**	**p** **<** **0.001**

BMI, Body mass index; BMR, Basal metabolic rate.

**Table 2:** Changes of bioelectrical impedance analysis at the end of the study.

Item	Preop	12-months postop	*p* value
BMR J/(h·kg)	8570.9 ± 1821.3	6834.70 ± 1451.3	*p* < 0.001
Total body fat mass (%)	47.7 ± 5.3	29.3 ± 8.4	*p* < 0.001
Total body fat mass (kg)	58.6 ± 13.7	23.0 ± 9.1	*p* < 0.001
Total free fat mass (%)	64.2 ± 13.6	54.6 ± 11.2	*p* < 0.001
Visceral fat level	15.9 ± 3.9	5.8 ± 2.7	*p* < 0.001
Total body water (%)	47.1 ± 9.9	51.8 ± 6.1	*p* < 0.001
Total body water (kg)	47.0 ± 9.9	39.9 ± 8.1	*p* < 0.001
Total muscle mass (kg)	60.9 ± 12.9	51.8 ± 10.7	*p* < 0.001
Right leg fat mass (%)	49.3 ± 7.3	31.9 ± 9.5	*p* < 0.001
Right leg free fat mass (kg)	11.6 ± 1.6	9.7 ± 2.1	*p* < 0.001
Right leg muscle mass (kg)	10.9 ± 2.5	9.2 ± 1.9	*p* < 0.001
Left leg fat mass (kg)	49.2 ± 7.6	32.5 ± 9.3	*p* < 0.001
Left leg free fat mass (kg)	11.5 ± 2.7	9.4 ± 1.9	*p* < 0.001
Left leg muscle mass (kg)	10.9 ± 2.6	8.9 ± 1.9	*p* < 0.001
Right arm fat mass (kg)	56.0 ± 8.3	34.9 ± 9.4	*p* < 0.001
Right arm free fat mass (kg)	3.4 ± 0.8	2.7 ± 1.0	*p* < 0.001
Right arm muscle mass (kg)	3.2 ± 0.9	2.5 ± 0.7	*p* < 0.001
Left arm fat mass (kg)	56.9 ± 8.1	35.4 ± 9.5	*p* < 0.001
Left arm lean mass (kg)	3.6 ± 0.9	2.7 ± 0.8	*p* < 0.001
Left arm muscle mass (kg)	3.4 ± 0.9	2.6 ± 0.7	*p* < 0.001
Trunk fat mass (kg)	43.9 ± 5.1	25.9 ± 8.6	*p* < 0.001
Trunk fat mass (kg)	26.8 ± 6.0	10.8 ± 4.9	*p* < 0.001
Trunk free fat mass (kg)	34.0 ± 6.3	29.9 ± 5.7	*p* < 0.001
Trunk muscle mass (kg)	32.5 ± 6.1	28.7 ± 5.5	*p* < 0.001

**Table 3:** Percent variance at 0–12 months sorted based on highest to lowest changes.

Item	Percent variance 0–12 months %
Total body fat mass (kg)	−60.1
Visceral fat level	−63.7
Trunk fat mass (kg)	−59.3
BMI (kg/m2)	−36.5
Right arm fat mass (kg)	−38.4
Left arm fat mass (kg)	−38.5
Left arm free fat mass (kg)	−24.2
Total body fat mass (kg)	−39.2
Trunk fat mass (kg)	−41.0
Right leg fat mass (kg)	−59.5
Left arm muscle mass (kg)	−24.2
Left leg fat mass (kg)	−34.9
BMR J/(h·kg)	−19.9
Left leg free fat mass (kg)	−17.9
Left leg muscle mass (kg)	−17.8
Right leg free fat mass (kg)	−16.3
Waist circumference (cm)	−26.5
Right leg muscle mass (kg)	−16.2
Right arm muscle mass (kg)	−20.7
Total muscle mass (kg)	−14.8
Total body lean mass (kg)	−14.9
Total body water (kg)	−14.9
Right arm lean mass (kg)	−20.6
Hip circumference (cm)	−22.7
Trunk muscle mass (kg)	−11.7
Trunk lean mass (kg)	−11.7
Albumin (g/dl)	−0.71
Total body water (%)	−35.7

## Data Availability

The data that support the findings of this study are available on request from the corresponding authors.

## Additional Points

There will be a lot of changes in body composition after significant weight loss. Laparoscopic mini-gastric bypass (LMGB) surgery is one of the most common types of bariatric surgery in Iran. Bariatric surgery-induced weight loss reduces both fat mass and lean mass all over the body, with certain areas reducing faster than others. There will also be some dangerous nutritional deficiencies after LMGB. These deficiencies should be detected and corrected early to avoid postoperative adverse effects, excessive loss of lean body mass. Serum albumin is perhaps the most widely used laboratory measure of nutritional status after LMGB. This study demonstrates post-bariatric changes of body composition in a carefully selected Iranian obese population in a high-volume bariatric unit. This study demonstrates how body composition will change after this procedure. This article shows which body parts lose their fat mass (FM) and lean mass (LM) in comparison to others, and which ones need more time. This study also investigates the ability of serum albumin in early detection of the LMGB-induced malnutrition.
